# Comparison of the effectiveness of trauma-focused cognitive behavioral therapy and paroxetine treatment in PTSD patients: Design of a randomized controlled trial

**DOI:** 10.1186/1471-244X-12-166

**Published:** 2012-10-09

**Authors:** A Rosaura Polak, Anke B Witteveen, Rogier S Visser, Brent C Opmeer, Nienke Vulink, Martijn Figee, Damiaan Denys, Miranda Olff

**Affiliations:** 1 Academic Medical Center (AMC), Department of Anxiety Disorders, University of Amsterdam (UvA), Meibergdreef 5, Amsterdam, 1105 AZ, The Netherlands; 2 Academic Medical Center (AMC), Clinical Research Unit, University of Amsterdam, Meibergdreef 5, Amsterdam, 1105 AZ, The Netherlands; 3 The Netherlands Institute for Neuroscience (NIN), an institute of the Royal Netherlands Academy of Arts and Sciences, Meibergdreef 47, Amsterdam, 1105 BA, The Netherlands; 4 Arq Psychotrauma Expert Group the Netherlands, Nienoord 5, Diemen, 1112 XE, The Netherlands

**Keywords:** PTSD, TF-CBT, Paroxetine, Effectiveness, Cost-effectiveness

## Abstract

**Background:**

The two most common interventions for Posttraumatic Stress Disorder (PTSD) are pharmacological treatment with SSRIs such as paroxetine and psychological treatment such as Trauma-Focused Cognitive Behavioral Therapy (TF-CBT). International guidelines recommend trauma-focused psychological interventions for all PTSD patients as first-line treatment (NICE). However, no clear-cut evidence is available to support this recommendation.

**Methods/design:**

In order to compare pharmacological treatment (paroxetine) and psychological treatment (TF-CBT) in (cost-) effectiveness on the short and the long term, we will randomize 90 patients with chronic PTSD to either paroxetine (24 weeks) or TF-CBT (10–12 weeks). We will assess symptom severity and costs before and after the intervention with the Clinician Administered PTSD Scale (CAPS), the Clinical Global Impression Scale (CGI) and the Trimbos/iMTA questionnaire for Costs associated with Psychiatric Illness (TiC-P).

**Discussion:**

This study is unique for its direct comparison of the most commonly used psychological intervention (TF-CBT) and pharmacological intervention (paroxetine) on (cost-) effectiveness on the short and the long term. The anticipated results will provide relevant evidence concerning long-term effects and relapse rates and will be beneficial in reducing societal costs. It may also provide information on who may benefit most from which type of intervention. Some methodological issues will be discussed.

**Trial Registration:**

Dutch Trial registration: NTR2235

## Background

Approximately 80% of the Dutch population experiences at least one traumatic event during lifetime [1]. The risk for development of PTSD after psychological trauma is approximately 10% in the Netherlands [1] as well as in other countries like the US [2]. Higher rates in women than in men have been found in the general population, e.g. 12% versus 4.6% [1,3-5]. PTSD generally continues for long periods of time, with a median time to recovery in the range of 3 to 5 years [2]. The high chronicity, severity, and co morbidity of PTSD are associated with high levels of functional and psychosocial disability [6], but also with high health care costs and economic impact due to health care utilization and negative effect on personal income [7]. Several effective treatments, however, are available to reduce symptoms and lower these costs.

At present, pharmacological treatment with selective serotonin reuptake inhibitors (SSRIs) and psychological treatment with trauma-focused behavioral therapy, i.e. Trauma-Focused Cognitive Behavioral Therapy (TF-CBT) or Eye Movement Desensitisation and Reprocessing (EMDR) are recommended for treatment of PTSD [8,9]. These recommendations are supported by placebo-controlled trials [10-12] and meta-analyses [13,14], which demonstrate that these psychological treatments are effective in treating PTSD symptoms. Selective serotonin reuptake inhibitors (SSRIs) such as paroxetine [15,16], sertraline [17,18] and fluoxetine [19-21] have shown favorable results in placebo-controlled randomized clinical trials. The effectiveness of these SSRIs has also been confirmed in a meta-analysis [22] and supported by reviews [23,24]. Notwithstanding the evidence coming from these guidelines and clinical trials, the choice of treatment in common clinical practice is rather arbitrary and seems to partly depend on indirect and direct assumptions of the clinician, style of health service delivery and patient factors (e.g. [25]). Mellman et al. [26] estimated that in a community-based sample, 77% of the PTSD patients received pharmacotherapy. The preference for pharmacotherapy may be due to the relatively poorer availability and accessibility of trauma-focused behavioral therapy, because of more time-consuming procedures such as referral, waiting list problems and the short handedness of behavior therapists. Little is known, however, about the difference in effectiveness between pharmacological versus psychological treatments.

The only meta-analysis that systematically compared effect sizes of both psychotherapeutic and pharmacological treatments for PTSD showed a slight advantage of CBT compared to other treatments on observer-related total PTSD symptoms [27]. Caution is, however, required when comparing effect sizes of pharmacological to psychotherapy trials as in pharmacological trials with placebo comparison control, non-specific attentional effects may have a more modest impact than in psychological therapy trials with waiting list controls [9]. Some studies compared the effectiveness of pharmacotherapy and trauma-focused therapy directly, suggesting a better long-term outcome for trauma-focused therapy than pharmacotherapy. One study [28] found relapse of PTSD symptoms at 6 months follow-up in the paroxetine group but not in the CBT group, and another study [11] revealed 58% asymptomatic patients 6 months after the trauma-focused psychological therapy group, compared to none in the SSRI group. However, these studies are rather small (respectively N=21 and N=88) and long-term consolidation of more than 6 months follow-up of the effects of pharmacological treatment were not yet reported. Therefore, several important questions regarding effectiveness, duration of treatment and relapse rates remain unanswered. Placebo-controlled studies that investigated treatment duration indicate beneficial effects of more sustained treatment. Martenyi et al. [29] have found a 16.1% relapse rate in 12-week fluoxetine treatment, compared to 5.8% in 24-week fluoxetine treatment. Furthermore, a study of Londborg et al. [30] showed that more than half of 41% non-responders after short-term treatment of 12 weeks achieved responder status during prolonged treatment of at least 24 weeks with SSRIs. Other studies have shown that treatment continuation for more than 24 weeks does not further reduce the PTSD symptoms but yields lower relapse rates than placebo [31,32]. These findings support the notion that a short-term course of treatment with SSRIs may be inadequate [33].

The importance of an adequately powered and designed trial determining whether trauma-focused psychological interventions differ from pharmacological interventions in terms of effectiveness and cost-effectiveness was already emphasized by national and international guidelines [8,9]. Gaining more knowledge on treatments and the consolidation of treatment effects will not only be beneficial for PTSD patients but may also reduce societal costs. Therefore we will compare pharmacological treatment with paroxetine and psychological treatment with TF-CBT in PTSD. We choose to compare these particular treatments as up to now, the majority of the empirical literature on psychotherapies for PTSD has focused on TF-CBT and this is repeatedly shown to be effective (e.g. [14,34,35]). Furthermore, particularly the SSRI paroxetine has proven to be effective in the reduction of symptoms from all three PTSD symptom clusters: re-experiencing, hyperarousal and avoidance [15,16] and has shown to be more effective than sertraline and fluoxetine in reducing PTSD symptom severity [23].

### Research aims and hypotheses

The aim of the proposed study is to compare the effectiveness and cost-effectiveness of Trauma-Focused Cognitive Behavioral Therapy (TF-CBT) to paroxetine in patients with PTSD in a randomized controlled trial in terms of PTSD symptom reduction. Secondary outcome measures include general measures of psychological wellbeing (i.e. anxiety and depression), quality of life, and related costs. Furthermore, we will take into account treatment responses by gender, age, and socio-economic status (incl. ethnic and cultural background) as previous studies show that women are twice as likely as men to develop PTSD during their lifetime [2] and indicate that different psychobiological mechanisms may play a role in the development of PTSD in women compared to men [5].

Based on previous findings of the effects of both treatments on clinical symptomatology over time, we expect the TF-CBT treatment to be more effective in PTSD symptom reduction than paroxetine most prominently at long-term follow-up, i.e. several months or years after cessation of both treatments. Furthermore, we hypothesize that CBT will be cost-effective, especially related to less expected relapse rates in comparison with pharmacological treatment. We will take into account direct medical costs (health care utilization as well as to inpatient and outpatient mental health care, day-treatment and primary physician care), direct non-medical costs (travel to and from health care providers, out-of-pocket costs) and indirect costs (lost productivity due to sick leave).

## Methods/design

The study is funded by the Netherlands Organization for Health Research and Development (ZonMw, grant no. 80-82310-98-09034). The study has been approved by the medical ethical board of the Academic Medical Center (AMC) (registration no: 09/080) and is conducted in accordance with the principles of the Declaration of Helsinki. The trial has been registered in the Dutch trial register and can be found at http://www.trialregister.nl (NTR2235).

### Study design

**Figure 1:**
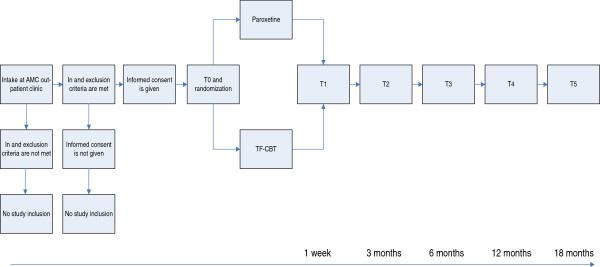
Flowchart.

The study is designed as a randomized controlled trial comparing TF-CBT and paroxetine treatment. The randomization and allocation procedure will be performed by a researcher who has no further role in data collection. The study design was set up with one pre-treatment assessment and four post-treatment assessments (at 1 week, 6 months, 12 months and 18 months) but before starting data-collection the authors added an assessment at 3 months as well (see Figure 1). All assessments will be performed by research workers blinded for the allocated treatment. The study will be performed at the department of psychiatry of the Academic Medical Center (AMC) in Amsterdam. Patients will be recruited over the course of 3 years, starting November 2009.

### Participants

Our study population will consist of patients with PTSD that are referred to the outpatient psychiatric clinic of the AMC. Inclusion criteria are: patients that fulfill all criteria for a diagnosis of chronic PTSD based on the DSM-IV [36], have a score of 45 or higher on the Clinician Administered PTSD Scale (CAPS [37]) 18 years of age or older, give written informed consent and are eligible for exposure therapy. Exclusion criteria are: suicidal risk; presence of any of the following DSM-IV diagnoses: psychotic disorder incl. schizophrenia, a bipolar disorder, depression with psychotic features, or excessive substance related disorder over the past 2 months, a primary diagnosis of severe depressive disorder; an organic disorder that interferes with either TF-CBT or paroxetine treatment; intolerance to paroxetine or any other SSRI; taking psychotropic medications that interact with paroxetine or no mastery of Dutch, English, Turkish or Arabic. Furthermore, female patients of childbearing potential must have a negative pregnancy test. Patients will receive a reimbursement that acknowledges the participants’ time and effort (15€ for each measurement as well as travel expenses).

### Sample Size

#### Sample size calculation

To compare the effectiveness of both interventions, the difference in CAPS scores between the two groups will be analyzed. We expect differences in effectiveness and cost-effectiveness between both treatments to be most prominent at follow-up, i.e. 6 months, 12 and 18 months after cessation of the 12-week treatment periods and therefore sample size calculations should ideally be based on expected differences at follow-up. However, due to very few data available for long-term power calculations, we initially choose to be conservative in our sample size calculation. Based on an earlier RCT on the efficacy of CBT and another RCT on the efficacy of paroxetine in the treatment of PTSD, we expect to find a total CAPS score of 23.7 (SD=26.1) in the cognitive behavioral group [38] and of 34.8 points (SD=25.7) in the paroxetine group [15]. Power calculations showed that a total of 89 participants in each treatment group will be required to demonstrate a difference of 11.1 points (alpha=5%, power=80%). To allow for 20% attrition at follow-up, we will assess 234 patients (N=117 in each treatment group).

#### Sample size calculation adjustment

We decided to adjust the sample size calculation and to optimize the power of the trial given the current study design, resulting in a smaller but more feasible sample size, in the light of possible slow recruitment. The original sample size calculation was based on the outcome on a single follow-up measurement, and was therefore rather conservative. As repeated measurements during follow-up provide additional data, differences can be estimated with higher accuracy, and therefore a smaller sample size is required to demonstrate a similar difference.

The increase in power does not solely depend on the number of measurements, but also on the correlation between measurements on the same patient (intra class correlation or ICC). Additional measurements add relatively less information to previous measurements if measurements are highly correlated. The required sample size for our study was therefore based on the study of Rochon [39] showing that for five measurements on each patient, for ICCs from 0 to 0.5, between 36 and 68 patients are required in each arm with an effect size of Cohen’s *d*=0.3, while between 14 and 25 patients per arm are needed with an effect size of 0.5. With an anticipated effect size of 0.46 we would need between 20 and 36 (depending on the actual ICC) patients per measurement per arm, respectively to gain a difference of 12 points on the CAPS at 18 months post-treatment. Anticipating possible attrition of study participants of 20%, we aim for 45 patients in each arm for the entire study period. Our new sample size is in line with another comparative study on long-term treatment effects of SSRI versus trauma-focused psychological treatment up to date that indicates that a smaller sample size may be sufficient to demonstrate a difference at 6-month follow-up [11].

### Materials

#### Interventions

**Psychotherapy Trauma-Focused Cognitive Behavioral Therapy (TF-CBT)** Participants allocated to the TF-CBT intervention, will receive 12 weekly sessions with a certified therapist who strictly follows a clinician manual for TF-CBT. CBT will be given by therapists who have clinical experience and at least a Master’s degree in Clinical Psychology or in Medicine and should have followed the training for TF-CBT at our department, in order to properly execute the clinician manual. The therapist will receive supervision regularly (once a month) and all sessions will be tape-recorded.

Trauma-Focused Cognitive Behavioral Therapy (TF-CBT) will be based on the model originally developed by Foa for female victims of rape [40]. In TF-CBT exposure (both in vivo and imaginal exposure) and cognitive restructuring (examining and challenging dysfunctional thoughts) are the most prominent elements next to psycho-education and anxiety management (e.g. muscle relaxation or breathing retraining). Subjects will take part in a structured therapy that follows a strict protocol developed by Creamer et al. [41] and based on the assumption that mental health specialists should be well-trained in the use of exposure, and particularly imaginal exposure. The clinician manual of Creamer is further developed and adjusted by the research staff of the current study. The first sessions are devoted to establishing a therapeutic alliance, giving psycho-education, and teaching relaxation skills and will take 60 minutes. Anxiety management strategies may be introduced, which include physical (i.e. breathing control, relaxation techniques, aerobic exercise, reducing stimulants such as caffeine and nicotine), cognitive (i.e. thought stopping, distraction techniques, imagery) and behavioral interventions (for addressing other associated problems, such as sleep disturbance, assertion and communication deficits). Not all strategies need to be provided, but according to the specific client several can be selected. In addition, the theoretical rationale of exposure-based therapy is introduced and repeated throughout treatment. Furthermore, the principle of the Subjective Units of Distress Score (SUDS), a rating system on a 100-point scale ranging from 0 (no anxiety) to 100 (extreme anxiety), is introduced and targets for exposure are identified. The following sessions imaginal exposure will be done. During imaginal exposure, the patient retells the traumatic experience in detail, SUDS are being registered and “hot spots” identified until anxiety reduction occurs. Exposure sessions can be accompanied by cognitive restructuring and focus on identifying, challenging and replacing maladaptive thought and beliefs associated with the trauma. The number of exposure sessions depends on the severity of the distress and the decrease of anxiety and will be 90 minutes. Homework assignments include practicing muscle relaxation or breathing techniques, confronting feared but safe situations (in vivo exposure), and daily listening to a taped narrative of the trauma (imaginal exposure). At the final sessions treatment progress is evaluated and relapse prevention is provided, reviewing the techniques used in therapy, evaluating their helpfulness, and discussing general termination issues. The relapse prevention session will be 60 minutes.

Each session, the therapist documents mental health status with the Clinical Global Impression scale. All subjects will be asked to weekly fill out the Impact of Event Scale-Revised [42].

##### Pharmacotherapy paroxetine (Seroxat)

Participants allocated to the pharmacotherapy intervention will receive paroxetine (Seroxat). Treatment will be given by certified psychiatrists according to a manual that is developed and adjusted by the research staff of this study.

Pharmacotherapy will be in accordance with Dutch guidelines of pharmacotherapy for anxiety disorders [8] and the NICE guidelines for clinical excellence [9]. Paroxetine treatment will start with psycho-education and discuss paroxetine intake and possible side effects. Paroxetine treatment will be initiated at 20 mg daily for 4 weeks. After 4 weeks the study psychiatrist can increase the dosage with increments of 10 mg daily each 4 weeks up to a maximum of 60 mg daily if according to the judgement of the study psychiatrist, the patient does not respond to lower dosages and if clinically tolerated. After 24 weeks of treatment the antidepressant will be gradually discontinued (tapering off in 10 or 20 mg decrements per week). If discontinuation/withdrawal symptoms do emerge and are mild, the psychiatrist will reassure the patient that these symptoms are not uncommon after discontinuing an antidepressant and will disappear in a few days. If symptoms are severe, reintroduction of the original antidepressant and gradual tapering is required.

On each visit during treatment the psychiatrist monitors for adverse side effects, evaluates compliance with the Morisky Medication Adherence Scale [43] and documents mental health status with the Clinical Global Impression scale. All subjects will be asked to fill out the Impact of Event Scale-Revised [42]. Every session takes 20–30 minutes. The psychiatrist will be instructed not to perform any direct psychotherapeutic interventions at the visits.

### Assessments

Assessments will take place pre-intervention (T0), 1 week post-intervention (T1) and 3, 6, 12 and 18 months at follow-up (T2-T5). Below we will describe the instruments that we will use in more detail.

### Clinical assessments

*Clinician Administered PTSD Scale (CAPS)* is one of the most widely used structured clinical interviews for diagnosing PTSD according to DSM-IV [44] and assessing PTSD symptom severity [37]. The CAPS distinguishes between the estimated frequency (range: 0–4) and intensity (range: 0–4) of the various symptoms. Frequency and intensity scores are added up to a total CAPS score (range: 0–136). The Dutch translation of the CAPS exhibits adequate validity and reliability. The internal consistency of this scale is good with alpha .63 for re-experiencing, .78 for avoiding and .79 for hyperarousal and .89 for all core PTSD symptoms together [45].

*Clinical Global Impression scale (CGI)* will be used to assess the response rate by rating the proportion of responders with a CGI improvement rating of “very much improved” or “much improved”. The CGI was first developed for use in psychopharmacology trials as part of the NIMH collaborative study of schizophrenia [46]. Since then it has been used as a standard primary outcome measure in studies investigating the efficacy of pharmacological treatments. Criteria for response will be a 30% or greater change from baseline on the CAPS and a final CGI rating of 1 or 2 (“much improved” or “very much improved”).

*M.I.N.I. International Neuropsychiatric Interview-Plus (M.I.N.I.-Plus)* will be used to assess psychopathology [47]. The M.I.N.I.-Plus is a widely used structured clinical interview that can diagnose past and present DSM-IV psychiatric disorders, such as mood disorders (i.e. major depressive, dysthymic or manic disorder), anxiety disorder (i.e. panic disorder, generalized anxiety disorder or obsessive compulsive disorder) or substance related disorders. Every module consists of screening questions which, if responded positively, will lead to additional examination for diagnosing the specific disorder. The M.I.N.I-Plus has reasonable to good interrater reliability (i.e. .84 for major depressive disorder and .43 for drug dependence). A Dutch translation of the M.I.N.I.-Plus is available [48].

### Self-report assessments

*Basic demographic variables*, i.e. birth date, sex, marital status, number of children, origin of parents, highest level of education, profession, number of working hours will be assessed.

*Trimbos/iMTA questionnaire for Costs associated with Psychiatric Illness (TiC-P)* is used to document resource utilization to estimate direct (i.e. medical consumption) and indirect costs (e.g. work absenteeism) associated with psychiatric illness [49]. Volumes of resource utilization will be valued by unit costs estimated according to the Dutch guideline on (unit) costing in healthcare [50]. GP visits, medical specialist visits, physical therapists, and travelling will be valued based on the guideline prices. Psychotherapy sessions will be based on reported expenses or recommended prices from the professional associations. Medication costs will be valued by their market prices [51]. The friction cost method will be used to estimate the duration of lost productivity, age adjusted average daily wages will be used to value this duration.

*World Health Organization-Quality of Life Bref (WHOQOL)* assesses quality of life [52]. The WHOQOL is a 26-item questionnaire to measure quality of life, including physical health, psychological health, social relationships and environment. Items are scored on 5-point scales from 1 (worse outcome) to 5 (best outcome) with a total range from 4–20. The Dutch version has demonstrated good content validity, construct validity and reliability [53].

*Euroqol 6-Dimensions scale (EQ-6D)* is an easy to apply self-administered questionnaire for describing and valuing quality of life [54] and can be used to generate cross-national comparisons of health state. The first part measures five health dimensions: mobility (MO), self-care (SC), daily activities (DA), pain/discomfort (PD), mood (MD) consisting of both anxiety and depression and cognitions (CD). In the EQ-6D patients report: 0 (no problems), 1 (moderate problems), and 2 (extreme problems). The second part (EQ-VAS) is a thermometer-like scale, in which patients rate their overall wellbeing from 0 (worst imaginable overall health) to 100 (best imaginable overall health). It originated from the ED-5D [55] that is shown to be valid and frequently used to assess generic quality of life and health status [56].

*Treatment preference* will be assessed by a question concerning of the preferred treatment if they would have had a choice. Preferred treatment can be indicated as: ‘TF-CBT’, ‘paroxetine’ or ‘no preference’.

*Hospital Anxiety and Depression Scale (HADS)* will be used to assess the level of depression and anxiety symptoms [57]. It is a well-established 14-item scale containing two subscales: HADS-A (Anxiety, 7 items, range: 0–21) and HADS-D (Depression, 7 items; range: 0–21). The HADS is validated in Dutch and showed satisfactory test-retest reliability for both subscale scores (Pearson’s r’s = .89 and .86 respectively) and validity [58].

*Life Events Checklist (LEC)* is a semi-structured interview that will be assessed to record the number and severity of the trauma(s) [59].

*Impact of Event Scale-Revised (IES-R)* is a questionnaire to assess the level of trauma reactions in the preceding 7 days that are associated with a traumatic event [42]. The IES-R contains 22 items (with 5-point Likert scales, 0–4) and three subscales, corresponding to the three symptom clusters in the DSM-IV PTSD diagnosis: re-experiencing (8 items, scale range 0–32), avoidance (8 items, range 0–32) and hyperarousal (6 items, range 0–24). The IES-R has been translated in Dutch and validated [60].

*Smoking screen* Standard Questionnaire Smoking (‘Standaardvraagstelling Roken’) is a questionnaire containing two questions that assess smoking habits. These questions have been used primarily by Dutch health organizations (GGD) to indicate smoking habits in elderly patients but are currently used to indicate smoking habits according to frequency and the variability (i.e. cigars, cigarettes) in other groups as well.

*Alcohol Use Disorders Identification Test (AUDIT)* was developed by the World Health Organization (WHO) in 1982 and is a screening instrument that assesses excessive drinking patterns and consists of 10 questions concerning recent alcohol use, alcohol dependence symptoms and alcohol-related problems [61]. This instrument is validated and designed for international use and cross-nationally standardized. The AUDIT identifies alcohol use as well as possible dependence, is brief, rapid and flexible and is consistent with the ICD-10 definitions of alcohol dependence and harmful alcohol use. Moreover, in several studies high validity as well as high reliability was found.

*Subjective Health Complaints Inventory (SHC)* is a 29-item scale that registers occurrence, intensity, and duration of subjective somatic and psychological complaints experienced during the past 30 days, without reference to specific diagnostic categories [62,63]. The intensity of each complaint graded on a 4-point scale (not at all/little/some/severe). An SHC total score is created by adding the scores of each item on somatic and psychological complaints. It records ailments based on objective diseases, but is particularly sensitive to health complaints with minimal or no clinical findings. The instrument can be divided into five categories: musculoskeletal pain, pseudo neurology, gastrointestinal problems, allergy and flu. The scores for each item range from 0 to 3, giving a total score from 0 (excellent) to 87 (very poor). The questionnaire has satisfactory validity and reliability.

*Morisky Medication Adherence Scale (MMAS)* is a self-reported measure of medication taking developed from a previously validated 4-item scale and supplemented with additional items addressing the circumstances surrounding adherence behavior [42].

### Procedure

All patients referred to the outpatient psychiatric clinic at the AMC will receive a diagnostic medical intake procedure, in which psychiatric and medical history are examined. In case PTSD diagnosis is suspected, the intake procedure will consist of standardized diagnostic measures for PTSD and other DSM-IV diagnoses (CAPS and M.I.N.I.-Plus). If PTSD is confirmed and all inclusion criteria are met, the patient will be requested to participate in the study. After giving informed consent, a baseline assessment (T0) will be performed. This assessment will consist of self-reported symptoms of PTSD, depression and anxiety and questionnaires on quality of life and costs. Subsequently, patients will be randomized to either TF-CBT or paroxetine treatment. One week after treatment, another assessment will be done (T1), consisting of the same questionnaires. At 3 months (T2), 6 months (T3), 12 months (T4) and 18 months (T5) assessments will be repeated. Figure 1 shows a flowchart of the study. Table 1 explores an overview on all instruments being assessed.

**Table 1:** Overview of instruments per assessment time point

	***Baseline (T0)***	***Treatment***	***1-week follow-up (T1)***	***3-month follow-up (T2)***	***6-month follow-up (T3)***	***12-month follow-up (T4)***	***18-month follow-up (T5)***
**Instruments**							
*Clinical instruments*							
CAPS	X		X	X	X	X	X
CGI	X	X	X	X	X	X	X
M.I.N.I.- Plus	X		X	X	X	X	X
*Self-report instruments*							
TiC-P	X		X	X	X	X	X
WHOQOL	X		X	X	X	X	X
EQ-6D	X		X	X	X	X	X
Preference	X						
HADS	X		X	X	X	X	X
LEC	X		X	X	X	X	X
IES-R	X	X	X	X	X	X	X
Smoking Screen	X		X	X	X	X	X
AUDIT	X		X	X	X	X	X
SHC	X		X	X	X	X	X
MMAS		X					

*CAPS*: Clinician Administered Posttraumatic Stress Disorder Scale (Blake et al., 1995); *CGI*: Clinical Global Impression scale (Guy, 1976); *M.I.N.I*.-Plus: MINI International Neuropsychiatric Interview-Plus (Sheehan et al., 1998); *TiC-P*: Trimbos/iMTA questionnaire for Costs associated with Psychiatric illness (Hakkaart-Van Roijen et al., 2002); *WHOQOL*: World Health Organization Quality of Life (WHOQOL GROUP, 1998); *EQ-6D*: Euoqol 6-Dimensions (Hoeymans et al., 2005); *HADS*: Hospital Anxiety and Depression Scale (Zigmond & Snaith, 1983); *LEC*: Life of Events Checklist; *IES-R*: Impact of Event Scale-Revised (Weiss & Marmar, 1997); *AUDIT*: Alcohol Use Disorders Identification Test; *SHC*: Social Health Complaints Inventory (Ursin et al., 1988; Eriksen et al., 1999); *MMAS*: Morisky Medication Adherence Scale (Morisky et al., 2008).

### Statistical analyses

All analyses will be performed according to the intention-to-treat (ITT) principle. Patients will be classified as responders and non-responders to treatment. Criteria for treatment response will be a 30% or greater change from baseline on the CAPS and a final CGI rating of 1 or 2 (“much improved” or “very much improved”).

Patients will also be classified in terms of compliance with the treatment regime and completion of the treatment and study procedures. Completers are those patients who finished the study scheme and who were available for at least one post-intervention assessment. Compliance concerns the adherence of treatment protocol, i.e. taking medication. Non-compliant patients are those patients who failed to take their medications or who failed to adhere to the TF-CBT sessions for >25% of the time. Regarding drop-out and loss to follow-up, a careful procedure will be followed in order to avoid an early exclusion of non-completers. Furthermore, adequate methods for imputation of missing data will be used, such as Maximum Likelihood Estimation (MLE) or Multiple Imputation method (MI).

Descriptive statistics will be used to examine differences in demographic, trauma-related characteristics at baseline between the TF-CBT group and the paroxetine group. Continuous CAPS scores at post-intervention assessments will be compared using analysis of covariance with study treatment (SSRI or TF-CBT) as main effect, adjusting for differences at baseline and other potential confounders (i.e. gender, age). Differences between both treatment groups will be evaluated by analysis of repeated measures, using linear mixed models. In multivariate analyses we will adjust the estimated differences for potential confounding variables (i.e. gender, age, SES, co-existence of a moderate depression). Regarding drop-out and loss to follow-up, different analytical approaches (complete case analysis, last observation carried forward, multiple imputation, best/worst outcome) will be followed in order to assess their impact on the study results. All analyses will be performed using SPSS 18.0.

#### Subgroup analyses

Several subgroup comparisons are of interest. Because of the power of the current proposal it will, however, not be possible to establish potential interaction effects or differential effects with statistical certainty. Therefore subgroup comparisons will have a hypothesis generating function. Since certain types of trauma and PTSD are more prevalent in females, non-whites, persons with low socio-economic status and in early adulthood (i.e., 18–22) [64], three more post-hoc analyses will be performed to examine the comparative effectiveness of both CBT and paroxetine in 1) men and women, 2) different age groups and 3) low and high socio-economic status.

### Economic evaluation

The economic evaluation will be performed from a societal perspective, with the costs per unit improvement on the primary outcome (CAPS score) as the primary outcome measure. The appropriate type of economic evaluation is conditional on the results [65]. We hypothesize that a more effective intervention will be associated with less health care utilization as well as less burden to relatives (time costs) and absence from paid work (productivity costs). Therefore, the primary analysis in the economic evaluation will be a cost-effectiveness analysis that evaluates costs associated with an improved PTSD outcome in terms of CAPS scores. In addition, a secondary analysis will evaluate cost differences in relation to differences in quality-adjusted life years (QALYs). This cost-utility analysis, resulting in an incremental cost-effectiveness ratio expressed in costs per QALY, will be included to allow comparison with other health-related interventions or programs. With a study horizon of 18 months, no discounting will be applied.

We will differentiate between direct medical, direct non-medical and indirect costs. Direct medical costs are associated with health care utilization related to the pharmacological or psychological treatment, as well as to inpatient and outpatient mental health care, day-treatment and primary physician care. Direct non-medical costs are generated by travel to and from health care providers. Indirect costs are associated with lost productivity due to absence from paid work.

Health state utilities to estimate QALYs will be derived from an EQ-6D measurement at baseline, as well as the follow-up assessments. Utility values for EQ-6D scores will be based on UK-estimates [53]. Utility scores will be uniformly interpolated, assuming constant health state between subsequent assessments.

Robustness of the results for uncertainty in the assumptions will be evaluated in sensitivity analyses, including: Dutch health states [66] and a linear interpolation between EQ-6D measurements, varying unit costs for pertinent volumes of health care utilization (e.g. therapy costs, productivity costs). In model-based analyses using data from literature about these middle to long-term effects and costs associated with PTSD patients we will extrapolate the results to estimate the effectiveness and cost-effectiveness for both treatment options on long term (3–5 years).

## Discussion

This RCT represents a unique study that aims to directly compare the most commonly used psychological intervention (TF-CBT) and pharmacological intervention (paroxetine) on (cost-) effectiveness in PTSD patients on the short and the long term. Although there is ample evidence that both treatments are effective, a systematic head to head comparison on the short term and especially on the long term is lacking. Thus far, only a handful of studies have compared pharmacological and psychological treatments directly [11,28] and there is uncertainty on the comparative longer term effectiveness and cost-effectiveness of these treatments.

Gaining insight in effectiveness of treatment in terms of reduction of PTSD symptoms will reduce significant personal suffering and help to reduce societal costs (i.e. regain of productivity and reduce sick leave) not only during treatment but also on the long term.

### Strengths and limitations

A particular strength of the study is that it is unique in comparing a pharmacological treatment with psychological treatments on the short and the long term. Its sample size is sufficient to differentiate the treatment in effectiveness and cost-effectiveness over the short and the long term, where at present only studies are available that did not take into account long-term effects [11,28].

Some limitations may affect the trial that need to be considered. Firstly, even though we aimed to reduce different influencing factors between pharmacological factors and psychological factors, this is rather difficult as the nature of the two treatments differs. Regarding this difference, the two treatments vary in duration as well as in number and length of the sessions, with pharmacological treatment approximately 24 weeks and psychological treatment consisting of approximately 12 weekly sessions. As we already outlined previously, the length of pharmacological treatment is very essential with possible high relapse rates during short-term treatments [29,31]. Despite these differences, we strived for similarity within the protocols as much as possible; both protocols consist of comparable psycho-education as well as a relapse prevention session. Furthermore, questionnaires will be performed in the same order in both treatment protocols. Other differences intertwined in the nature of both treatments should not be eliminated since the comparison of both treatments should be based on conditions that reflect routine clinical practice in this pragmatic effectiveness trial.

Another possible limitation may lie in the fact that we only include PTSD patients that are able to receive monotherapy with either TF-CBT or paroxetine. Some patients may not be eligible for exposure or monotherapy with pharmacotherapy due to a variety of reasons, such as the presence of primary severe depressive disorder or suicidal ideation, other comorbid disorders (i.e. personality disorders) and severe psychosocial problems that may interfere with treatment. Patients with more severe PTSD symptoms are often recommended combination therapy with both pharmacotherapy and psychotherapy [33]. A recent meta-analysis [67] comparing the effectiveness of combination therapies with the separately delivered interventions, could however not draw clear conclusions due to lack of evidence and urged for large randomized controlled trials. Despite the fact that combination therapies are common, and direct comparison leads to exclusion of patients with more complex symptoms, we nonetheless think it is very important to first carefully investigate the relative superiority of one treatment to another. By directly comparing these separate treatments we will be able to draw solid conclusions on (cost-) effectiveness.

### Implications for practice

Clinicians’ decisions about optimal care, and the clinical practice guidelines that inform these decisions, will of course rely to a large extent on evidence from adequate RCTs. For example, recently, a growing number of studies on the effectiveness of Eye Movement Desensitization and Reprocessing (EMDR) have been conducted (i.e. [10]) as well as meta-analyses (i.e. [34]) and reviews (i.e. [68]), comparing results across RCTs on EMDR and other treatments, showing that EMDR is equally effective as TF-CBT (see also Nijdam et al. [12]). This finding led to recommendation and implementation of EMDR as a first-line psychological treatment besides TF-CBT. Likewise, evidence on the direct comparison of effective psychological and pharmacological interventions regarding (cost-) effectiveness is scarce and this RCT may help answering questions concerning the most optimal care. If our premise that TF-CBT is superior to paroxetine in terms of sustainable effectiveness and cost-effectiveness for the treatment of PTSD, this could have major implications for current clinical practice.

## Competing interests

The authors declare that they have no competing interests.

## Authors' contributions

ARP and ABW drafted the manuscript. ARP, RSV, ABW, NV, MF, DD and MO contributed to the development of the intervention protocols and implementation of the interventions. BCO contributed to the design of the economic evaluation as well as the statistical procedures. All authors contributed to editing the manuscripts and read and approved the final manuscript.

## Pre-publication history

The pre-publication history for this paper can be accessed here:

http://www.biomedcentral.com/1471-244X/12/166/prepub
